# Intake of *Koji Amazake* Improves Defecation Frequency in Healthy Adults

**DOI:** 10.3390/jof7090782

**Published:** 2021-09-21

**Authors:** Atsushi Kurahashi, Toshihiko Enomoto, Yoshifumi Oguro, Ayana Kojima-Nakamura, Kazuya Kodaira, Kenichi Watanabe, Nobuhiro Ozaki, Hiroshi Goto, Masao Hirayama

**Affiliations:** 1 Hakkaisan Brewery Co., Ltd., 1051 Nagamori, Minamiuonuma City, Niigata 949-7112, Japan; toshihiko.enomoto@hakkaisan.jp (T.E.); y.oguro@hakkaisan.jp (Y.O.); ayana.nakamura@hakkaisna.co.jp (A.K.-N.); k.kodaira@hakkaisan.jp (K.K.); 2 Department of Endocrinology and Metabolism, Niigata University Graduate School of Medical and Dental Sciences, 754 Ichibancho, Asahimachi-dori, Chuo-ku, Niigata City, Niigata 951-8510, Japan; wataken@med.niigata-u.ac.jp; 3 Niigata Medical Association of Occupational Health Inc., 1185-3 Kitaba, Nishi-ku, Niigata City, Niigata 950-1187, Japan; tomiyama@niwell.or.jp; 4 Niigata Bio-Research Park Inc., 316-2 Higashijima, Akiha-ku, Niigata City, Niigata 956-0841, Japan; goto@nbrp.co.jp (H.G.); hirayama@nbrp.co.jp (M.H.)

**Keywords:** *amazake*, *Aspergillus oryzae*, defecation, intestinal microbiota, *koji*

## Abstract

Reportedly, the intake of *koji amazake*, a beverage made from steamed rice fermented by *Aspergillus oryzae*, improves defecation frequency. However, its functional ingredients and mechanism of action remain unclear. To compare the effects of *koji amazake* and a placebo beverage on defecation frequency and to identify the functional ingredients and mechanism of action, a randomized, placebo-controlled, double-blind parallel-group comparative trial was performed on two groups. The *koji amazake* had 302 ± 15.5 mg/118 g of *A. oryzae* cells, which was not in the placebo. Compared with the placebo group, the *koji amazake* group showed a significant increase in weekly defecation frequency at 2 weeks (5.09 days vs. 4.14 days), 3 weeks (5.41 days vs. 4.18 days), and 4 weeks (5.09 days vs. 3.95 days), along with an increase in the weekly fecal weight at 4 weeks (724 g vs. 501 g). The intake of *koji amazake* did not induce significant intergroup differences in the fecal SCFA concentration, whereas it significantly decreased the relative abundance of *Blautia* and significantly increased that of *Bacteroides* at 3 weeks. Therefore, *koji amazake* intake improved defecation frequency, and *A. oryzae* cells played potentially important roles as functional ingredients.

## 1. Introduction

Several fermented beverages and food products consumed globally are produced by the action of microorganisms and/or their enzymes. Fermented foods form a significant part of diets worldwide and typically constitute approximately one-third of the global food intake [1]. Fermentation not only improves food preservation, nutritional value, and quality, but also benefits human health by facilitating the growth of probiotic strains in foods. Probiotics are viable microorganisms that beneficially affect their hosts, including humans, by improving their intestinal microbial balance [2]. One of the other expected functions of probiotics is the improvement of defecation frequency. Yogurt, a representative fermented food product, contains lactic acid bacteria and bifidobacteria, which reportedly improve defecation frequency in healthy individuals. The intake of live *Lactobacillus casei* Shirota and *L. brevis* KB290 improved the defecation frequency and stool quality by enhancing short-chain fatty acid (SCFA) production by intestinal microflora [3,4]. A similar effect was reported for *Bifidobacterium longum* BB536 [5]. Probiotics for improving human defecation have mostly been limited to lactic acid bacteria and bifidobacteria, although a few other food microorganisms have also been considered.

Recently, two human studies reported the effect of *koji amazake* on stool defecation. *koji amazake* is a non-alcoholic, white colored, Japanese traditional sweet beverage made from steamed rice fermented by *Aspergillus oryzae* [6]. The raw materials are rice-*koji* and water. It becomes sweetened when saccharification enzymes such as α-amylase and glucoamylase of *A. oryzae* break down rice starch in rice-*koji* into glucose in a saccharification process at 50–60 °C. Therefore, *koji amazake* includes rice, *A. oryzae*, and its metabolites. In one study, the defecation frequencies of female college students (*n* = 12) in *koji amazake* intake and non-intake groups were compared, and a statistically significant difference was found in the intragroup comparison from baseline to 12 days post-intake [7]. The second study showed that in the comparison of defecation frequencies between two groups with the intake of *koji amazake* and its mixture with *Lactobacillus sakei* UONUMA, the defecation frequency was significantly higher in the *koji amazake* group [8]. However, both reports lacked scientific evidence, including information on the functional ingredients and mechanism of action underlying the increase in defecation frequency, which are essential parameters for the Japanese approval system of Foods for Specified Health Use [9]. Probiotics are widely known to improve gastrointestinal health through the following mechanisms: modification of gastrointestinal microbiota, production of probiotic metabolites, including prebiotics, and production of SCFAs [10]. Prebiotics and probiotics are known to increase the defecation frequency [11]. Prebiotics are non-digestible food ingredients, such as non-digestible fiber and oligosaccharides, with beneficial effects, such as selective stimulation of colonic bacterial species [12].

We have previously reported that *koji amazake* contains over 300 compounds, including the fermentation metabolites of *A. oryzae* [13]. In particular, *koji amazake* contains various glucooligosaccharides, such as maltose, nigerose, kojibiose, trehalose, sophorose, gentiobiose, maltotriose, isomaltotriose, panose, and raffinose [14]. These glucooligosaccharides contain prebiotic components, such as gentiobiose (Glc(β1-6)Glc) [15], raffinose (Gal(α1-6)Glc(β1-2)Fru) [16], isomaltooligosaccharides (IMOs) (including isomaltose (Glc(α1-6)Glc), isomaltotriose (α1-6)Glc(α1-6)Glc), and panose (Glc(α1-6)Glc(α1-4)Glc)), which reportedly modify the intestinal microbial composition and improve gastrointestinal conditions [17,18]. In addition, rice-*koji*, the raw material for *koji amazake*, contains monohexosylceramides composed of two sugar moieties: *N*-2′-hydroxyoctadecanoyl-l-*O*-β-d-glucopyranosyl-9-methyl-4,8-sphingadienine and *N*-2′-hydroxyoctadecanoyl-l-*O*-β-d-galactopyranosyl-9-methyl-4,8-sphingadienine [19,20,21]. Reportedly, these *koji* ceramides serve as prebiotics for *Blautia coccoides* [22]. *koji amazake* also contains *A. oryzae* cells. It has been reported that chitin-glucan prepared from the cell wall of *Aspergillus niger* induces specific changes in intestinal bacteria and further increases the bacterial metabolites in feces, including butyric, iso-valeric, caproic, and vaccenic acids [23]. Therefore, *koji amazake* fulfills the required conditions for classification as a synbiotic, which contains multiple potential prebiotic candidates for improving defecation frequency. However, only a limited number of studies are available on the functional ingredients and mechanisms of action.

This was a randomized, placebo-controlled, double-blind parallel-group comparative trial, in which rice syrup was used as a placebo. The primary outcome was the defecation frequency per week, and secondary outcomes were changes in fecal characteristics, fecal SCFA content, and fecal microbiota composition. The information obtained was used to determine the active functional ingredients of *koji amazake* and the mechanism of action.

## 2. Materials and Methods

### 2.1. Test Beverages

Commercial *koji amazake* (Hakkaisan Brewery Co., Ltd., Minamiuonuma, Japan) and rice syrup (prepared using hydrolyzing enzymes without fermentation with *A. oryzae*, as previously described [14]) (118 g/bottle) were used as the test beverages. The nutritional content per bottle of *koji amazake* and placebo are shown in Table 1. The energy, protein, lipid, carbohydrate, available carbohydrate, dietary fiber, ash, and moisture contents were measured using the official method adopted by the Japan Food Research Laboratories (Tokyo, Japan), as previously described [24].

### 2.2. Quantification for A. oryzae and Its Metabolites in Koji Amazake

Contents of oligosaccharides, glycosylceramides (GlcCer), and *A. oryzae* cells were quantified in order to reveal functional components in *koji amazake*. The levels of glucose and other saccharides were measured as previously described (*n*= 3) [13,14].

The standard GlcCer from *A. oryzae* was prepared at a facility at the Foundation for Promotion of Material Science and Technology of Japan (Tokyo, Japan). Ten grams of freeze-dried *A. oryzae* mycelium was homogenized with 250 mL of methanol and then with 500 mL of chloroform. Next, 200 mL of water was added, and the mixture was filtered for 1 min using a filter paper with a separating funnel and then allowed to stand overnight. The organic solvent phase was dried using an evaporator; 10 mL of KOH-methanol solution, 0.5 mL of chloroform/methanol (2:1, *v*/*v*), and 7.5 mL of water were added; and a saponification was performed for 3 h at 37 °C. The saponified sample was mixed with 16 mL of chloroform, 8 mL of methanol, and 6 mL of water. The extraction process described above was performed three times. Next, the organic solvent phase was collected and evaporated. The dried sample was dissolved in methanol and was then subjected to silica gel chromatography. The sample was washed using chloroform/methanol (99:5, *v*/*v*). Afterwards, a fraction containing GlcCer was eluted using chloroform/methanol (90:10, *v*/*v*) and evaporated. The lipid fraction containing GlcCer was separated precisely using an HPLC system equipped with a fraction collector to obtain two species of GlcCer, which were used as GlcCer standards for *A. oryzae*. GlcCer from rice was purchased as a commercial product (Nagara Science, Gifu, Japan). The GlcCer content in *koji amazake* and placebo was measured using ACQUITY UPLC H-Class with ACQUITY QDa Detector (Waters Corporation, Milford MA, USA). GlcCer was separated using the ACQUITY UPLC BEH C18 column (2.1 mm × 150 mm, 1.7 μm) (Waters Corporation, Milford MA, USA). Solvent A was 10 mM ammonium formate, and solvent B was 10 mM ammonium formate–methanol. The duality mobile phase gradient used for analysis was as follows: 0 min, 3% A, 97% B; 15–19 min 100% B; 20–25 min 3% A, 97% B. The flow rate was 0.2 mL/min. The other parameters adopted were as follows: column temperature, 40 °C; injection volume 1 μL. Each GlcCer content of *koji amazake* (*n* = 17) and placebo (*n* = 3) is shown in Table 1.

With slight modification from a previous study [25], the amount of *A. oryzae* cells in *koji amazake* (*n* = 17) and placebo (*n* = 3) was estimated by quantifying N-acetylglucosamine (GlcNAc). Beverage sample (5 g) was centrifuged and supernatant was discarded. The pellet was washed 3 times with 50 mM phosphate buffer (pH 7.0). In order to degrade chitin in the sample, 10 mL of 50 mM phosphate buffer containing 10 mg Yatalase (Takara Bio Inc, Japan) was added to the pellet, and then incubated for 1 h at 37 °C. Quantification of GlcNAc was followed by the method proposed by Reissig et al. [26]. The reaction mix containing 500 μL of the sample and 100 μL of 0.8 M potassium tetraborate (pH 9.0) was boiled for 3 min at 100 °C and then quickly cooled. The *p*-dimetylaminobenzaldehyde (DMAB) solution (10 g of DMAB was dissolved 12.5 mL 12 N HCl and adjusted to 100 mL by adding acetic acid) was diluted 9-fold with acetic acid. Cooled sample was mixed with 3 mL of diluted DMAB solution and incubated for 20 min at 37 °C. After the sample was cooled, absorbance was measured at 585 nm. In this study, 1 mg mycelium was estimated to be 139 μg GlcNAc based on a previous report [25].

### 2.3. Study Population

From 60 applicants, 44 healthy adults with 2–5 days of weekly defecation were recruited at the Niigata Bio-research Center. The adults were screened according to the inclusion and exclusion criteria. Individuals with screening test results and questionnaire results appropriate for this study, as judged by an investigator, were included. The exclusion criteria were as follows: (i) individuals with diabetes and under medical treatment; (ii) individuals with diseases (e.g., liver, kidney, heart, blood diseases, and infections requiring notification); (iii) individuals with a history of gastrectomy, enterectomy, and gastrointestinal diseases, judged to be inappropriate for this study; (iv) pregnancy, women with chances of pregnancy, and lactating women; (v) participants in other clinical trials; and (vi) individuals with concerns judged to be inappropriate for this study by an investigator.

### 2.4. Study Design

The study had a randomized, placebo-controlled, double-blind parallel-group comparative design. It was conducted between October 2019 and May 2020 at the Niigata Bio-research Center (Niigata, Japan). Randomization was performed in a stratified manner based on the weekly defecation day (days/week) at the screening visit. The study period spanned 6 weeks, consisting of a non-intake observation period (−1 to 0 w), intake period (0 to 3 w), and follow-up period (4 to 5 w). The test beverage was administered once daily during the intake period. The participants were instructed to avoid the consumption of other *amazake* drinks and similar beverages and the introduction of changes to their daily activities, including diet and exercise. After the participants provided written informed consent and their screening procedures were completed, they were recruited to the study. The participants were randomly assigned to two groups: *koji amazake* and placebo.

During the study, all participants were instructed as follows: avoid an irregular lifestyle, including overeating and poor sleep; avoid introducing changes to routine lifestyle, including diet, sleep, and exercise; do not donate blood; do not start intake of new health foods; and contact the study staff if experiencing any signs or symptoms of changes in health conditions. During the test period starting from the day before testing, the participants were prohibited from drinking alcohol, over-exercising, overeating, and sleeping insufficiently on the day before testing. From 20:00 h on the day before testing, the consumption of food and beverages, except water, was prohibited.

### 2.5. Physiological and Biochemical Variables of the Participants

The following physiological and biochemical variables of the participants were measured during the screening visit using standard methods: height; body weight; body mass index; body fat percentage; systolic and diastolic blood pressure; levels of aspartate aminotransferase, alanine aminotransferase, γ-glutamyl transpeptidase, high-density and low-density lipoprotein cholesterol, triglyceride, blood urea nitrogen, creatinine, fasting blood glucose, white blood cells, red blood cells, hematocrit, hemoglobin, and platelets. These measurements were performed at the Clinical Laboratory Division of the Association of Occupational Health, Inc. (Niigata, Japan).

### 2.6. Questionnaires on Defecation and Collection of Fecal Samples

We instructed the participants to fill out questionnaires on their defecation patterns and characteristics and to collect their fecal samples during three different periods. Questions were asked on the defecation frequency (number of days), defecation frequency (number of times), fecal quantities (in terms of the number of Japanese standard chicken eggs, shown schematically), and fecal characteristics (in terms of shape, color, odor, and sensation after defecation). The participants observed their fecal characteristics, recorded the scores each day throughout the 6-week study period, and submitted the weekly records to the study staff. Each fecal sample was collected by the participants in a designated container from the TechnoSuruga Laboratory Co., Ltd. (Shizuoka, Japan) on the last day of the non-intake observation period (0 w), intake period (3 w), and follow-up period (5 w). The samples were delivered to the TechnoSuruga Laboratory under refrigeration within 24 h of defecation.

### 2.7. Defecation Patterns and Fecal Characteristics

Using the data collected from the questionnaires, the defecation frequency (number of days/week and number of times/week), fecal weight (g/week), and fecal characteristic scores (for shape, color, odor, and sensation after defecation) for each week were calculated during the 6 weeks of the study period. The fecal weight was estimated by counting the number of Japanese standard chicken eggs (50 g) approximately representative of the fecal volume, as reported by Ogata et al. [27], and a fecal specific gravity of 1.0 [28]. The fecal characteristic scores were calculated based on the scores selected by the participants from five optional scores (1–5) against each characteristic in reference to the Bristol stool form scale [29]. The five optional scores were as follows: fecal shape, 1 (very hard), 2 (hard), 3 (smooth), 4 (soft), and 5 (watery); fecal color, 1 (black), 2 (dark brown), 3 (brown), 4 (ocher), and 5 (yellow); fecal odor, 1 (very strong), 2 (strong), 3 (moderate), 4 (almost no odor), and 5 (odorless); and sensation after defecation, 1 (very unrefreshing), 2 (unrefreshing), 3 (general), 4 (refreshing), and 5 (very refreshing).

### 2.8. Fecal pH and SCFA Concentration

Measurements of fecal pH and SCFA concentration were performed at the TechnoSuruga Laboratory Co., Ltd. After sampling, the pH was measured immediately using a digital pH meter (LAQUAtwin B-712, HORIBA, Ltd., Kyoto, Japan). For measuring the organic acid content, 0.1 g of each fecal sample was placed in a 2.0 mL tube with zirconia beads and suspended in Milli-Q ultrapure water. The samples were heated at 85 °C for 15 min, vortexed at 5 m/s for 45 s using FastPrep-24 (MP Biomedicals, Santa Ana, CA, USA), and centrifuged at 15,350× *g* for 10 min. The supernatant was filtered using a 0.2 μm filter (Ultrafree-MC PTFE LG; Merck Millipore, Danvers, MA, USA) and analyzed for the detection of nine fatty acids: succinic acid, lactic acid, and SCFAs (formic acid, acetic acid, propionic acid, *iso*-butyric acid, *n*-butyric acid, *iso*-valeric acid, and *n*-valeric acid) using an organic acid analysis system (Shimadzu Corp., Kyoto, Japan) with a Prominence^TM^ high-performance liquid chromatography system, a conductivity detector (CDD-10A (Shimadzu)), two columns arranged in tandem (Shim-pack SCR-102(H) (300 mm × 8 mm ID)), a guard column (Shim-pack SCR-102(H) (50 mm × 6 mm ID)), a mobile phase (5 mM *p*-toluenesulfonic acid), and a reaction solution containing 5 mM *p*-toluenesulfonic acid, 100 μM ethylenediaminetetraacetic acid, and 20 mM bis(2-hydroxyethyl)iminotris(hydroxymethyl)methane. The flow rate and oven temperature were 0.8 mL/min and 45 °C, respectively. The limits of quantification were 0.05 mg/g for succinic acid, lactic acid, acetic acid, and propionic acid and 0.10 mg/g for formic acid, *iso*-butyric acid, *n*-butyric acid, *iso*-valeric acid, and *n*-valeric acid, respectively.

### 2.9. DNA Extraction and Next-Generation Sequencing (NGS) Analysis of Bacterial Flora in Feces

Genomic DNA was extracted from the fecal samples, and NGS analysis was performed at the TechnoSuruga Laboratory Co., Ltd. Fecal solids in the suspension were ground with zirconia beads using a FastPrep-24 Instrument (MP Biomedicals) at 5 m/s for 2 min. Bacterial DNA was extracted from 200 μL of the suspension using an automated DNA extraction machine (GENE PREP STAR PI-480, Kurabo Industries Ltd., Osaka, Japan) according to the manufacturer’s instructions. The DNA content measured using a NanoDrop™ ND-8000 (NanoDrop Technologies, Wilmington, DE, USA), and the final concentration (10 ng/μL) of the DNA sample was noted.

NGS analysis of bacterial 16S rRNA gene (16S rDNA) was performed using MiSeq (Illumina, San Diego, CA, USA), as previously reported by Takahashi et al. [30]. Briefly, the V3–V4 hypervariable regions of 16S rDNA were amplified using 341F and 806R, the universal primers for bacteria, and touchdown PCR was performed using a GeneAmp PCR system 9700 (Applied Biosystems, Foster City, CA, USA) [30,31]. Sequencing was performed using a paired-end method and modified to a 2 × 300 bp cycle run using an Illumina MiSeq sequencing system (Illumina, San Diego, CA, USA) and MiSeq Reagent Kit version 3 (600 cycle) chemistry. Paired-end sequencing was performed with a read length of 301 bp [32]. After demultiplexing, a clear overlap was observed in the paired-end reads. The sequences were subjected to quality checks and filtering using the FastX toolkit [33], and only reads having >99% of their sequence with quality value scores ≥ 20 were extracted for further analysis. The chimeric sequences were eliminated using the USEARCH software [34,35]. Based on the sequences, the taxonomic positions were identified at 97% similarity using the Metagenome@KIN analysis software (World Fusion, Tokyo) and the TechnoSuruga Lab Microbial Identification database (DB-BA 13.0; TechnoSuruga Laboratory), which only lists bacteria with standing in the taxonomic nomenclature [31,36,37].

### 2.10. Statistical Analysis

Results are presented in terms of the mean values and standard deviations. Inter-group comparisons between the *koji amazake* and placebo groups were performed using repeated measures analysis of variance (ANOVA), followed by an unpaired *t*-test. Intra-group differences in the values at weeks 3 and 5 vs. those at week 0 (baseline) were compared using Dunnett’s multiple comparisons test. Values with *p* < 0.05 were considered statistically significant, and those with *p* < 0.1 were considered to be trending toward significance. Statistical analyses were performed using the EZR software (version 1.27; Saitama Medical Center, Jichi Medical University, Saitama, Japan).

## 3. Results

### 3.1. Analysis of Functional Components Derived from A. oryzae

To reveal functional components in *koji amazake* manufactured by *A. oryzae*-derived fermentation process, we intensively investigated contents of oligosaccharides, GlcCer, and *A. oryzae* cells as its candidates. Furthermore, these candidates are reported to have the ability to modify the intestinal microbiome [18,22,23]. The nutrient composition of the test beverages (*koji amazake* and placebo) is shown in Table 1. The beverages only had minor differences with respect to the nutritional values of proteins, fats, and carbohydrates. In fact, the prebiotic oligosaccharide content in *koji amazake* (3.12 ± 0.03 g) was only 1.26 times greater than that in the placebo (2.47 ± 0.07 g). Although raffinose and gentiobiose have been reported to be contained in *koji amazake* [13], these oligosaccharides were not detected in the present study. Some prebiotic oligosaccharides, such as isomaltose and kojibiose, were produced by the transglycosylation of α-glucosidase. In particular, panose and kojibiose was contained at only low level, but these oligosaccharides were specifically produced by *A. oryzae*. Conversely, the oligosaccharides present in the saccharified solution were primarily IMOs derived from the branched chain of amylopectin. The GlcCer was localized on plasma membrane and plays essential physiological roles in fungi [22]. *A. oryzae*-derived GlcCer have unique C_9_-metylated spingoid base that was not contained in rice [19]. *koji amazake* had a GlcCer content of 1.39 ± 0.12 mg, which was 3.4 times greater than that in the placebo (0.41 ± 0.04 mg). The GlcCer content comprised two types of *A. oryzae*-derived GlcCer (Figure S1A,B) and six types of rice-derived GlcCer (Figure S1C–H). The chemical structures of GlcCer have also been reported earlier [21,38,39]. *A. oryzae*-derived GlcCer was not present in the placebo. The galactosylceramide was not detected in the beverages. Main materials for *koji amazake* are rice-*koji* which is steamed rice fermented by *A. oryzae*. Therefore, *A. oryzae* cells (302 ± 15.5 mg) were detected in *koji amazake*. Conversely, *A. oryzae* cells were not detected in placebo due to the enzymatic manufacturing process.

### 3.2. Participants and Their Background Characteristics

All 44 participants (18 males and 26 females) successfully completed this trial without any drop-outs, and data from all participants were included in the final analysis. The results of the hematological and biochemical tests conducted at the screening visit were within the normal range, and the representative background characteristics of the 22 participants in each group are summarized in Table S1. The weekly defecation frequency values were 3.95 ± 1.02 days/week and 4.68 ± 1.55 times/week in the *koji amazake* group and 4.00 ± 1.04 days/week and 4.73 ± 1.63 times/week in the placebo group. There were no significant differences (*p* = 0.773 and 0.927, respectively) in the background characteristics between the two groups, as determined using an unpaired *t*-test. No adverse events were reported during the 6-week study period.

### 3.3. Defecation Patterns and Fecal Characteristics

**Figure 1:**
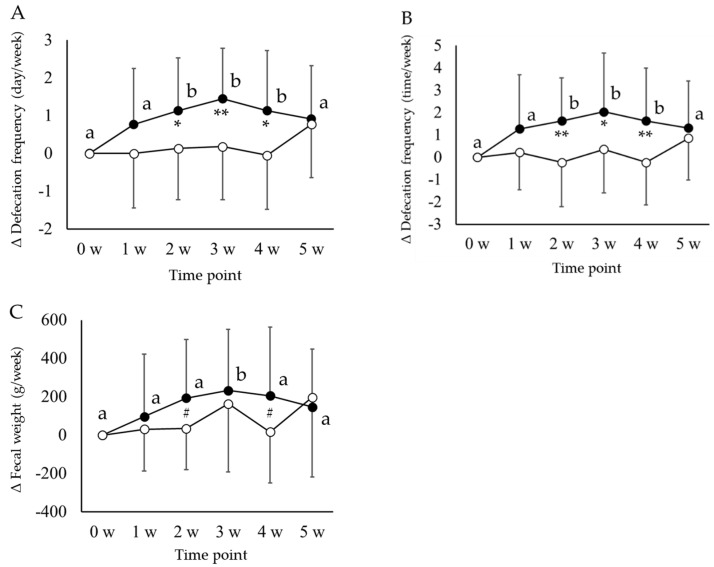
Comparison of the changes (Δ) in weekly defecation frequencies ((**A**) days per week and (**B**) times per week) and fecal weight ((**C**) g per week) from baseline values (0 w) in the *koji amazake* group (filled circle) and placebo group (empty circle). Each value is expressed as mean ± standard deviation. Inter-group comparisons between the *koji amazake* and placebo groups were performed using repeated measures analysis of variance (ANOVA) followed by a paired *t*-test; significant differences are indicated by ** *p* < 0.01, * *p* < 0.05, and # *p* < 0.1. a, b Intra-group changes from baseline values (0 w) in each group at six time points were compared using Dunnett’s multiple comparisons test; values with different superscript letters in each profile indicate significant difference (*p* < 0.05).

Based on the data recorded in the questionnaires, the weekly values of defecation patterns (frequency and weight) and fecal characteristics of participants from the *koji amazake* and placebo groups during the 6-week study period are summarized in Table 2, along with the results of the statistical analysis of inter- and intra-group comparisons of the two groups. The number of weekly defecation days, defecation frequency, and fecal weight increased significantly in the *koji amazake* group compared with the placebo group, as evaluated using repeated measures ANOVA followed by an unpaired *t*-test at each time point. Weekly defecation days and frequency of *koji amazake* group increased significantly at 2 w (*p* = 0.037 and 0.004), at 3 w (*p* = 0.010 and 0.035), and at 4 w (*p* = 0.027 and 0.009), compared with the corresponding values of the placebo group. Weekly fecal weight of the *koji amazake* group increased significantly at 4 w (*p* = 0.028) and showed a trend to increase at 2 w (*p* = 0.063). In terms of changes (Δ) from baseline (0 w) till the five time points, the Δdefecation frequency (number of days), Δdefecation frequency (number of times), and Δfecal weight also increased significantly in the *koji amazake* group compared with the placebo group. The values obtained were as follows: weekly Δfrequency (number of days) at 2 w (1.14 ± 1.39 days vs. 0.14 ± 1.36 days, *p* = 0.020), 3 w (1.45 ± 1.34 days vs. 0.18 ± 1.40 days, *p* = 0.004), and 4 w (1.14 ± 1.58 days vs. 0.08 ± 1.43 days, *p* = 0.013); weekly Δfrequency (number of times) at 2 w (1.64 ± 1.92 times vs. −0.23 ± 1.92 times, *p* = 0.003), 3 w (2.05 ± 2.63 times vs. 0.36 ± 1.97 times, *p* = 0.021), and 4 w (1.64 ± 2.36 times vs. −0.23 ± 1.90 times, *p* = 0.006); and weekly Δfecal weight at 2 w (194 ± 305 g vs. 34 ± 214 g, *p* = 0.052 (a trend)) and 4 w (206 ± 358 g vs. 17 ± 268 g, *p* = 0.054 (a trend)) (Figure 1).

To evaluate the effects of *koji amazake* during intake and after termination of intake, the intra-group changes from baseline (0 w) to 3 and 5 w were compared using Dunnett’s multiple comparisons test. The values in the *koji amazake* group increased significantly (presented as items (time point, value vs. value at 0 w; *p* value)): weekly frequency (number of days) (3 w, 5.41 ± 1.53 days vs. 3.95 ± 1.05 days; *p* = 0.025), weekly frequency (number of times) (3 w, 6.73 ± 2.76 times vs. 4.68 ± 1.59 times; *p* = 0.025), and color (2 w, 3.11 ± 0.38 and 3 w, 3.21 ± 0.49 vs. 2.76 ± 0.40; *p* = 0.032 and 0.003, respectively). However, the significant differences observed initially were not observed after 5 weeks of follow-up. The placebo group showed no significant intra-group differences in defecation patterns or fecal characteristic scores at 3 or 5 w.

### 3.4. Fecal pH and SCFA Concentration

The fecal pH and fatty acid concentration in the *koji amazake* and placebo group were measured on the last day of each study period during the non-intake observation period (0 w), intake period (3 w), and follow-up period (5 w), and the following pH values were obtained: *koji amazake* group, 7.02 ± 0.49, 6.97 ± 0.64, and 7.09 ± 0.43; placebo group, 7.19 ± 0.57, 7.00 ± 0.37, and 7.1 ± 0.42, respectively. The pH values of fecal samples from the *koji amazake* and placebo groups at 3 w were lower than those at baseline (0 w) or 5 w; however, no statistically significant difference was observed in either inter-group or intra-group comparisons. At each time point, the fecal concentrations of succinic acid, lactic acid, formic acid, acetic acid, propionic acid, iso-butyric acid, *n*-butyric acid, *iso*-valeric acid, and *n*-valeric acid were measured using gas chromatography mass spectrometry (GC-MS), and the detection rates of these fatty acids in the participants throughout the study period were 72.7%, 11.4%, 1.5%, 100.0%, 100.0%, 53.8%, 99.2%, 75.0%, and 66.7%, respectively. Furthermore, the weekly value for the fecal fatty acid weight was calculated by multiplying the concentration with the corresponding weekly fecal weight. The results are shown in Table 3; the results for lactic acid and formic acid were not provided owing to the low detection rates (< 20%). No significant intergroup difference was observed in either concentration or weekly weight of each fatty acid at any time point. In the intra-group comparison, the total SCFA concentration increased significantly from baseline (0 w) to 3 w in the placebo group, but the increase in the *koji amazake* group was not significant. However, the weekly weights of total SCFA and total fatty acids increased significantly from baseline (0 w) to 3 w in both groups.

### 3.5. NGS Analysis of Bacterial Flora in Feces

**Figure 2:**
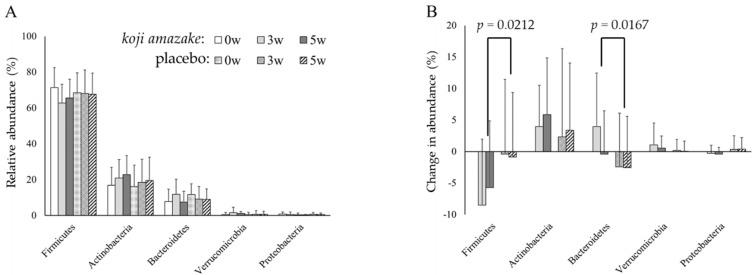
Inter− and intra−group comparison of relative abundances (**A**) at three time points (0, 3, and 5 w), and changes in the abundances of five dominant bacterial phyla (**B**) from baseline values (0 w) in the *koji amazake* and placebo groups. Each value is expressed as mean ± standard deviation. The *p* values indicate significant inter−group difference, analyzed using repeated measures ANOVA (*Firmicutes*: group *p* = 0.0146, time *p* = 0.0103, group × time *p* = 0.0282; *Bacteroidetes*: group *p* = 0.0516, time *p* = 0.164, group × time *p* = 0.0250) followed by a *t*-test. a, b Columns with different letters indicate significant difference (*p* < 0.05), with intra−group comparisons among three time points in each group of each phylum using Dunnett’s multiple comparisons test.

A total of 4,879,397 filtered high-quality sequence reads were generated, including the following (results are presented as time point, total reads of 44 samples, average ± SD per sample, max, and min): 0 w, 672,457, 15,283 ± 3890, 24,479, 10,338; 3 w, 2034.436, 46,237 ± 9.036, 77,124, 31,283; 5 w, 2,172,504, 49,375 ± 6759, 74,864, 34,688. Eight bacterial phyla were identified (Table S2). The dominant phyla were *Firmicutes*, *Actinobacteria*, *Bacteroidetes*, *Verrucomicrobia*, and *Proteobacteria*, and members of these five phyla constituted an average of 97.8% (range 97.3–97.8%) in both groups. As shown in Figure 2A, statistical analysis of the relative abundance of each phylum revealed no significant differences in the inter-group and intra-group comparisons. The *Firmicutes*-to-*Bacteroidetes* ratios of *koji amazake* and the placebo group were 26.7 ± 33.4 and 7.5 ± 4.0 at 0 w, 15.8 ± 28.3 and 32.5 ± 83.2 at 3 w, and 20.7 ± 30.7 and 48.1 ± 170.3 at 5 w, respectively. The difference of mean values with large standard deviations showed the ratios of participants in both two groups were spread out over a large range even at 0 w, and there was no inter- and intra-group significant difference. However, analysis of the inter-group comparison of the changes (Δ) in relative abundance from baseline (0 w) to 3 and 5 w showed significant differences in Δ*Firmicutes* (relative abundance) (−8.51% vs. −0.43%, *p* = 0.0212) and Δ*Bacteroidetes* (3.56% vs. 2.44%, *p* = 0.0167) at 3 w. Furthermore, the Δ*Firmicutes* value decreased significantly from baseline (0 w) to 3 w (*p* = 0.0033) in the intra-group comparison of the *koji amazake* group (Figure 2B).

**Figure 3:**
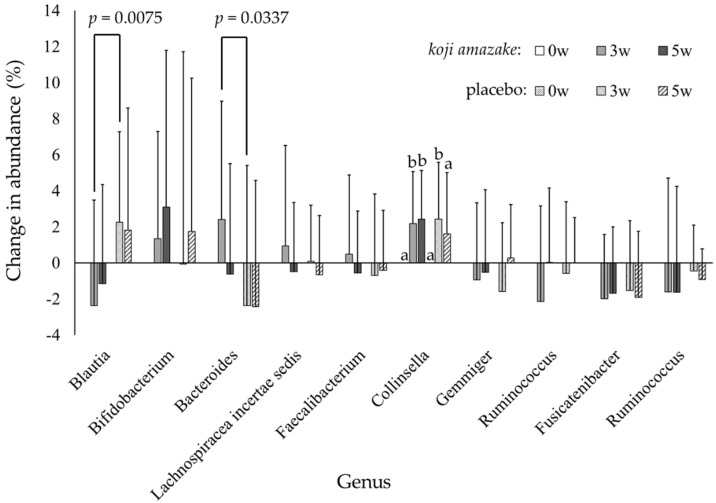
Inter− and intra−group comparison of the changes in Δ relative abundance from baseline values (0 w) to 3 w and in each of ten dominant bacterial genera of the *koji amazake* and placebo groups. Each value is expressed as mean ± standard deviation. Delta (Δ) relative abundance is the change in relative abundance compared with the value at baseline. Statistical analyses: *p* values indicate significant inter−group difference, analyzed using repeated measures ANOVA (*Blautia*: group *p* = 0.0193, time *p* = 0.8846, group × time *p* = 0.0276; *Bacteroidetes*: group *p* = 0.0830, time *p* = 0.2191, group × time *p* = 0.0617) followed by a *t*-test. a, b Columns with different letters indicate significant difference (*p* < 0.05), with intra-group comparison in each group of each genus at three time points, as calculated using Dunnett’s multiple comparisons test.

There were 229 genera with relative abundances more than 0.01% and 19–21 genera (8.3%–9.2% of all detected genera) with relative abundances more than 1.0%, depending on the time point of measurement and group. Table 4 shows the comparison of relative abundances of taxa in the *koji amazake* and placebo groups at the three time points (0, 3, and 5 w), with each bacterial genus showing a relative abundance greater than 1.0%. No significant difference was observed in the inter-group comparison (using repeated measures ANOVA followed by *t*-test) or intra-group comparison (using Dunnett’s multiple comparisons test) of each genus. However, the inter-group comparison of the change (Δ) in relative abundance from baseline (0 w) to 3 and 5 w showed significant differences, as shown in Figure 3. In the *koji amazake* group, a significant decrease was observed for Δ*Blautia* (3 w, –2.37% vs. 2.25%, *p* = 0.0075), and a significant increase was observed for Δ*Bacteroides* (3 w, 2.40% vs. 2.38%, *p* = 0.0337). Furthermore, the intra-group comparison revealed a significant increase in Δ*Collinsella* at 3 w both in the *koji amazake* and placebo groups, as determined using Dunnett’s multiple comparisons test.

## 4. Discussion

The findings of this study showed that the intake of *koji amazake including metabolites produced by A. oryzae*, a Japanese traditional fermented beverage, was beneficial for increasing the defecation frequency and fecal weight in healthy adults and also for changing the relative abundances of the genera *Blautia* and *Bacteroides* in the fecal microbiota.

The increase in the weekly defecation frequency (number of days and number of times) and weekly fecal weight in the *koji amazake* group was also significant in the intra-group comparison, whereas no significant intra-group changes were noted in the placebo group. However, the significant increase in the values in the *koji amazake* group for both comparisons was not observable at 5 w after the intake was stopped. With respect to the scores for fecal characteristics, including shape, color, odor, and sensation, no significant differences were observed in the inter-group or intra-group comparisons of the two groups, barring the brightening of the fecal color at 2 and 3 w, which resulted in a significant intra-group difference in the *koji amazake* group.

No statistically significant group difference was observed in fecal pH and fecal fatty acid concentrations (Table 3). The reasons for this are not completely understood, but we supposed the reason is attributed to the rapid absorption of SCFAs and an increase in fecal weight. SCFAs are the primary energy source for colonocytes and are rapidly absorbed by them [40]. The fecal SCFA concentration was not found to be affected directly by the consumption of prebiotics or probiotics [41,42]. In addition, the fecal SCFA concentration was reportedly affected by fecal characteristics, such as fecal weight and consistency; this was also observed upon the intake of mushrooms, which resulted in a significant increase in stool weight with no significant difference in fecal SCFA concentrations [43]. The rapid absorption of SCFA and an increase in the fecal weight also affected the SCFA concentrations. The total SCFA concentration in the *koji amazake* group was found to increase from baseline (0 w) to 3 w, but this increase was not significant. However, the weekly total SCFA weight at 3 w increased significantly compared with that at 0 w. These results suggest that some components of *koji amazake* may function synergistically and contribute to the increase in SCFA.

The 16S rRNA gene profiling revealed that *koji amazake* intake affected the relative abundances of several dominant taxa of the intestinal microbiota. No significant differences in the relative abundances of either phylum or genus were observed in the inter-group comparisons at each time point between the two groups. However, the inter-group comparison of the Δ relative abundance from baseline (0 w) to 3 w showed significant changes in dominant taxa. We anticipated an increase in the relative abundance of *Bifidobacterium* in both groups as compared with corresponding 0 w samples because both beverages contained several kinds of oligosaccharides reported as bifidogenic prebiotics, such as gentiobiose [15], raffinose [16], and isomaltooligosaccharides [17,18]. However, the Δ*Bifidobacterium* value did not differ significantly in either inter-group or intra-group comparison. We have previously reported that eight indigestible oligosaccharides are synthesized in *koji amazake* through *A. oryzae* fermentation [13]. However, the amounts of oligosaccharides known as prebiotics contained in *koji amazake* was 1.26-fold relative to that in the placebo (Table 1). The main oligosaccharide was isomaltose-derived branched chains of amylopectin in rice starch. Therefore, isomaltose in *koji amazake* and placebo were close to 1.96 g and 1.89 g, respectively. Isomaltooligosaccharaides have been reported to improve defecation frequency in constipated patients with intake of 10 g/day for 1 week [18]. Since the amount of isomaltose contained in *koji amazake* is less than that amount, other substances might be involved.

*Koji*, which is main raw material in *koji amazake*, contains GlcCer derived from both *A. oryzae* and rice. This study revealed *koji amazake* contained 3.4-fold GlcCer (1.39 ± 0.02 mg) relative to the placebo (0.41 ± 0.04 mg). This significant difference was certainly attributed to *A. oryzae* (Table 1). The GlcCer derived from plants is known to have an ability to improve skin barrier function at intake of 1.2–1.8 mg [44,45]. An in vitro test has reported that GlcCer extracted from *A. oryzae* improve skin barrier function through enhancement of the expression of OCLN encoding occluding, which is one of the proteins constituting tight junction [46]. Taken together, GlcCer is a functional substance that works at the mg level; however, further verification is needed to determine whether such a small amount of ceramide contributes to improved bowel movements.

*koji amazake* also contains 302 ± 15.5 mg of *A. oryzae* cells. Rodriguez et al. reported that chitin-glucan prepared from the cell wall of *A. niger* induces specific changes intestinal bacteria and further increases the bacterial metabolites in feces, including SCFA [23]. The cell wall of *A. oryzae* is the same as that of *A. niger*, which is composed of chitin, α-1,3-glucan, and β-1,6-branced β-1,3-glucan [47]. Therefore, alternation of relative abundance of *Blautia* and *Bacteroides* between intake of *koji amazake* and placebo was probably attributed to the cell wall of *A. oryzae* cells in *koji amazake*. We conclude that the intestinal microbiome modified by intake of *A. oryzae* cells contained in *koji amazake* mainly contributed to the improvement in defecation frequency. However, identification in the intestinal microbiome was only feasible up to the genus level. The precise effect of *koji amazake* on the intestinal microbiome should be investigated in a future study.

Interestingly, the *Firmicutes*-to-*Bacteroidetes* ratios at 3 w and 5 w became numerically lower in the *koji amazake* group than in the placebo group. The *Firmicutes*-to-*Bacteroidetes* ratio has been shown to be directly correlated with obesity in several studies, indicating that the ratio increases in individuals who are overweight and obese [48,49]. Recently, the *Firmicutes*-to-*Bacteroidetes* ratio was reported to decrease based on dietary intake, such as the intake of soy milk [50] or probiotics [51]. Manipulation of the ratio based on the alteration of dietary components is expected to serve as a potential therapeutic approach for the prevention and management of obesity. Therefore, *koji amazake* might be one of the dietary candidates with the potential to decrease the *Firmicutes*-to-*Bacteroidetes* ratio.

Several mechanisms have been reported to explain the improvement in defecation frequency following the intake of probiotics and prebiotics, such as enhancing colonic peristalsis by SCFA generation, increasing stool bulking by alteration of microbial composition, and promoting softness of feces [10]. The results of the present study strongly suggest that an increase in stool bulking by the alteration of the intestinal microbiota may improve the defecation frequency.

In conclusion, the results of this study reveal that the intake of *koji amazake* improved defecation by increasing stool bulking via the alteration of intestinal microbiota and that *koji amazake* is a beneficial beverage for individuals with a low defecation frequency.

## Figures and Tables

**Table 1:** Nutritional values of test beverages (per 118 g in a bottle).

Item	*koji amazake*	Placebo
Energy (Kcal)	127.4	128.6
Moisture (g)	86.4	85.6
Protein (g)	1.4	1.5
Fat (g)	0.4	0.2
Total GlcCer (mg)	1.39	0.41
GlcCer from A. oryzae (mg)	1.16	ND
GlcCer from rice (mg)	0.23	0.41
Ash (g)	0.1	0.1
Carbohydrate (CHO) (g)	29.7	30.6
Dietary fiber (g)	0.2	0.1
Available CHO (g)	29.5	30.5
Digestible CHO (g)	26.4	28.0
Glucose (g)	26.1	27.8
Maltose (g)	0.17	0.20
Trehalose (g)	0.14	ND
Prebiotics (g)	3.12	2.47
Isomaltose (g)	1.96	1.89
Isomaltotiose (g)	0.10	0.18
Panose (g)	0.10	ND
Sophorose (g)	0.66	0.32
Nigerose (g)	0.20	0.08
Kojibiose (g)	0.10	ND

**Table 2:** Weekly values and the statistical analysis of defecation patterns (frequency and weight) and fecal characteristics of a *koji amazake* group and placebo groups during a 6-week study period.

Group *n*	Time Point (Period) ^(1)^	Weekly Defecation Patterns ^(3,4)^			Scores of Fecal Characteristics ^(2,3,4)^			
		Defecation Days (Days/Week)	Frequency (Times/Week)	Weight (g/Week)	Shape	Color	Odor	Sensation
*koji amazake* *n* = 22 age range 20–66	0 w (−1 to 0 w)	3.95 ± 1.05 ^a^	4.68 ± 1.59 ^a^	518 ± 277	3.24 ± 0.49	2.76 ± 0.40 ^a^	2.89 ± 0.67	3.31 ± 0.88
	1 w (0 to 1 w)	4.73 ± 1.75 ^a^	5.95 ± 2.61 ^a^	616 ± 330	3.08 ± 0.51	3.01 ± 0.39 ^a^	2.96 ± 0.38	3.15 ± 0.78
	2 w (1 to 2 w)	5.09 ± 1.51 ^a,^*	6.32 ± 2.21 ^a,^*	712 ± 385 ^#^	3.02 ± 0.44	3.11 ± 0.38 ^b^	2.96 ± 0.43	3.42 ± 0.84
	3 w (2 to 3 w)	5.41 ± 1.53 ^b,^*	6.73 ± 2.76 ^b,^**	752 ± 360	2.97 ± 0.40	3.21 ± 0.49 ^b^	2.88 ± 0.42	3.31 ± 0.65
	4 w (3 to 4 w)	5.09 ± 1.80 ^a,^*	6.32 ± 2.53 ^a,^**	724 ± 402 *	3.11 ± 0.36	2.97 ± 0.45 ^a^	2.78 ± 0.45	3.20 ± 0.78
	5 w (4 to 5 w)	4.86 ± 1.55 ^a^	6.00 ± 2.60 ^a^	665 ± 387	3.11 ± 0.32	3.03 ± 0.41 ^a^	2.82 ± 0.38	3.43 ± 0.70
Placebo *n* = 22 age range 22–66	0 w (−1 to 0 w)	4.00 ± 1.07	4.73 ± 1.67	484 ± 235	3.34 ± 0.58	2.78 ± 0.54	2.86 ± 0.72	3.02 ± 0.85
	1 w (0 to 1 w)	4.00 ± 1.41	4.95 ± 2.03	515 ± 264	3.16 ± 0.54	3.04 ± 0.54	3.15 ± 0.54	3.17 ± 0.60
	2 w (1 to 3 w)	4.14 ± 1.42	4.50 ± 1.71	519 ± 272	3.15 ± 0.67	3.08 ± 0.60	3.01 ± 0.60	3.40 ± 0.71
	3 w (2 to 3 w)	4.18 ± 1.50	5.09 ± 9.18	647 ± 386	3.27 ± 0.70	3.06 ± 0.54	2.92 ± 0.56	3.15 ± 0.70
	4 w (3 to 4 w)	3.95 ± 1.46	4.50 ± 1.85	501 ± 225	3.31 ± 0.66	3.07 ± 0.51	2.86 ± 0.58	3.12 ± 0.73
	5 w (4 to 5 w)	4.77 ± 1.34	5.59 ± 1.94	681 ± 441	3.45 ± 0.64	3.04 ± 0.50	3.00 ± 0.50	3.26 ± 0.64
ANOVA *p* value ^(5)^	Group	0.0638	0.0463	0.2296	0.0886	0.9871	0.5117	0.4944
	Time	0.0003	0.0011	0.0000	0.1035	0.0001	0.0350	0.1135
	Group × time	0.0036	0.0031	0.0282	0.6584	0.7229	0.6205	0.8051

The values are the mean ± standard deviation. ^(1)^ Each time point was the last day of each period; a non-intake observation period (−1 to 0 w), intake period (0 to 1 w, 1 to 2 w, and (2 to 3 w), and follow-up period (3 to 4 w and 4 to 5 w). ^(2)^ Scores were calculated based on the scores selected from 5 optional scores described in Materials and Methods. ^(3)^ **, *, # Values within a column with asterisk indicate significant difference (** *p* < 0.01, * *p* < 0.05) and trends towards significance (# *p* < 0.1) with inter-group comparison between the corresponding time points by two-way repeated measures analysis of variance (ANOVA) followed by unpaired *t*-test. ^(4)^ a, b Values within a column with different superscript alphabets indicate significant difference (*p* < 0.05) with intra-group comparison among six time points by Dunnett’s multiple comparison test. ^(5)^ By repeated measures analysis of variance (ANOVA): group (between-subjects effect), time (within-subjects effects), group × time (interactions).

**Table 3:** Fecal fatty acid concentrations and weekly fecal fatty acid weights of *koji amazake* and placebo groups on the last day of non-intake observation, intake, and follow up periods. ^(1,2)^.

Fatty Acid	Group	Fecal Fatty Acid Concentration, μmol/g Feces			Weekly Fecal Fatty Acid Weight, mmol		
		0 w	3 w	5 w	0 w	3 w	5 w
Succinic acid	*amazake*	2.22 ± 5.44	1.98 ± 4.36	5.02 ± 9.74	0.56 ± 0.98	1.09 ± 2.51	1.44 ± 2.91
	Placebo	1.33 ± 0.92	2.86 ± 4.93	3.13 ± 6.16	0.52 ± 0.89	2.51 ± 3.02	1.73 ± 3.95
Acetic acid	*amazake*	41.3 ± 15.1	48.1 ± 17.2	47.0 ± 12.7	21.7 ± 15.3 ^a^	37.0 ± 27.3 ^b^	32.1 ± 23.4 ^a^
	Placebo	31.3 ± 15.8 ^a^	44.8 ± 16.4 ^b^	42.6 ± 19.4 ^a^	16.7 ± 14.8	30.3 ± 27.9	26.9 ± 19.7
Propionic acid	*amazake*	13.0 ± 5.5	15.9 ± 8.5	13.6 ± 5.5	7.29 ± 6.26	12.87 ± 11.08	9.18 ± 7.68
	Placebo	10.8 ± 4.1 ^a^	15.8 ± 7.0 ^b^	14.6 ± 7.5 ^a^	5.25 ± 3.37 ^a^	11.08 ± 10.65 ^b^	9.13 ± 6.40 ^a^
*iso*-Butyric acid	*amazake*	2.03 ± 0.92	1.37 ± 0.67	1.77 ± 0.85	0.37 ± 0.79	0.79 ± 0.84	0.65 ± 0.90
	Placebo	1.59 ± 0.59	1.80 ± 0.54	2.04 ± 0.77	0.28 ± 0.36 ^a^	0.84 ± 0.83 ^b^	0.53 ± 0.68 ^a^
*n*-Butyric acid	*amazake*	8.95 ± 5.13	10.18 ± 5.97	9.99 ± 5.88	4.42 ± 2.92 ^a^	7.83 ± 6.72 ^b^	6.71 ± 6.49 ^a^
	Placebo	6.98 ± 4.12	9.27 ± 4.71	10.05 ± 7.51	3.69 ± 3.88	6.72 ± 4.51	6.49 ± 6.22
*iso*-Valeric acid	*amazake*	2.68 ± 1.50	2.08 ± 0.79	2.18 ± 1.44	0.79 ± 1.31	1.35 ± 1.07	1.15 ± 1.30
	Placebo	2.18 ± 1.06	2.48 ± 1.15	2.39 ± 1.21	0.67 ± 0.52	1.07 ± 1.07	1.28 ± 1.07
*n*-Valeric acid	*amazake*	2.71 ± 2.57	2.18 ± 0.84	1.91 ± 0.82	0.78 ± 1.19	1.36 ± 1.26	1.11 ± 1.21
	Placebo	1.38 ± 0.39	2.08 ± 1.05	2.14 ± 0.93	0.35 ± 0.38 ^a^	1.26 ± 1.06 ^b^	0.73 ± 0.83 ^a^
Total SCFA	*amazake*	66.8 ± 24.8	78.4 ± 29.9	74.8 ± 18.9	35.3 ± 24.8 ^a^	61.2 ± 45.6 ^b^	50.9 ± 35.8 ^a^
	Placebo	52.2 ± 21.3 ^a^	73.9 ± 22.6 ^b^	71.5 ± 32.1 ^b^	26.9 ± 21.2 ^a^	41.4 ± 45.0 ^b^	45.0 ± 30.3 ^a^
Total fatty acids	*amazake*	74.3 ± 43.9	85.4 ± 43.9	78.8 ± 19.6	38.4 ± 28.7 ^a^	62.4 ± 46.4 ^b^	52.4 ± 35.3 ^a^
	Placebo	60.7 ± 27.8 ^a^	91.6 ± 45.3 ^b^	74.4 ± 32.1 ^a^	27.9 ± 22.9 ^a^	52.7 ± 44.3 ^b^	47.2 ± 31.6 ^a^

The values are the mean ± standard deviation, *n* = 22. ^(1)^ Feces were collected on the last day of the following periods; non-intake observation period (−1 to 0 w), intake period (0 to 3 w), and follow-up period (3 to 5 w). ^(2)^ a, b Values with different superscript alphabets within a row of each measuring item indicate significant difference (*p* < 0.05) with intra-group comparison with corresponding baseline (0 w) values by Dunnett’s multiple comparison test.

**Table 4:** Comparison of relative abundances at three time points (0 w, 3 w, and 5 w) of *koji amazake* and placebo groups in each bacterial genus with relative abundance more than 1.0%.

Genera	Relative Abundance%, *Koji Amazake n* = 22			Relative Abundance%, Placebo *n* = 22		
	0 w	3 w	5 w	0 w	3 w	5 w
*Blautia*	15.40 ± 8.62	13.03 ± 9.65	14.25 ± 7.64	12.17 ± 6.45	14.42 ± 6.07	14.42 ± 8.71
*Bifidobacterium*	13.57 ± 8.96	14.91 ± 8.72	16.66 ± 9.11	12.38 ± 10.57	12.31 ± 11.12	12.31 ± 12.03
*Bacteroides*	6.56 ± 5.77	8.96 ± 6.63	5.94 ± 5.28	9.52 ± 5.09	7.14 ± 6.16	7.14 ± 5.04
*Lachnospiracea* ^(a)^	6.50 ± 3.48	7.44 ± 5.45	6.01 ± 3.65	6.78 ± 4.22	6.86 ± 4.67	6.86 ± 3.19
*Faecalibacterium*	5.74 ± 4.60	6.23 ± 4.13	5.17 ± 3.39	6.18 ± 4.72	5.49 ± 4.50	5.49 ± 4.60
*Collinsella*	2.84 ± 2.97	5.01 ± 4.85	5.26 ± 4.96	3.15 ± 2.54	5.58 ± 4.11	5.58 ± 3.99
*Gemmiger*	5.36 ± 4.09	4.42 ± 3.43	4.83 ± 4.02	4.95 ± 5.18	3.36 ± 2.83	3.36 ± 5.81
*Ruminococcus*	5.42 ± 5.72	3.28 ± 4.01	5.43 ± 5.64	4.58 ± 5.63	4.01 ± 4.53	4.01 ± 4.86
*Fusicatenibacter*	4.66 ± 4.10	2.68 ± 2.40	2.97 ± 2.97	5.09 ± 4.13	3.57 ± 3.05	3.57 ± 2.83
*Ruminococcus2*	4.43 ± 6.23	2.82 ± 2.70	2.80 ± 2.48	3.99 ± 3.17	3.55 ± 4.01	3.55 ± 2.47
*Roseburia*	3.31 ± 3.80	2.31 ± 2.22	2.46 ± 1.91	3.00 ± 3.74	330 ± 3.86	3.30 ± 3.85
*Clostridium XlVa*	2.15 ± 1.65	2.56 ± 2.11	2.44 ± 2.49	2.61 ± 2.38	2.91 ± 2.35	2.91 ± 1.64
*Rejected hit*	2.28 ± 4.90	1.84 ± 1.87	2.10 ± 2.98	2.43 ± 3.25	2.18 ± 2.84	2.18 ± 2.26
*Anaerostipes*	3.21 ± 2.56	2.40 ± 2.27	2.35 ± 2.08	2.06 ± 1.96	1.56 ± 1.70	1.56 ± 1.56
*Streptococcus*	1.99 ± 3.37	1.41 ± 1.83	1.81 ± 1.94	1.79 ± 1.88	2.11 ± 2.14	2.11 ± 2.70
*Clostridium IV*	1.32 ± 2.14	1.02 ± 1.14	1.49 ± 1.92	2.85 ± 3.64	1.68 ± 2.51	1.68 ± 3.17
*Clostridium XVIII*	1.30 ± 1.90	1.41 ± 1.61	1.24 ± 1.17	1.13 ± 1.03	1.63 ± 1.53	1.63 ± 1.53
*Dorea*	1.59 ± 0.95	1.41 ± 1.73	1.39 ± 1.33	1.54 ± 1.76	1.50 ± 1.39	1.50 ± 1.34
*Megamonas*	0.04 ± 0.17	1.40 ± 4.46	0.43 ± 1.86	0.62 ± 2.61	1.49 ± 6.29	1.49 ± 8.45
*Megasphaera*	1.05 ± 2.48	1.37 ± 2.28	1.49 ± 2.66	0.38 ± 1.40	1.59 ± 6.59	1.59 ± 2.10
*Prevotella*	0.21 ± 0.55	1.30 ± 4.37	0.17 ± 0.45	0.17 ± 0.40	0.22 ± 0.64	0.22 ± 0.18
*Dialister*	0.88 ± 1.99	0.98 ± 1.59	1.35 ± 2.81	0.61 ± 0.89	0.97 ± 1.25	0.86 ± 1.40
*Clostridium XI*	0.51 ± 0.74	0.62 ± 0.69	0.75 ± 0.99	1.20 ± 1.04	0.72 ± 0.68	0.99 ± 1.40
*Coprococcus*	1.02 ± 1.56	0.69 ± 1.00	0.86 ± 1.26	0.94 ± 1.08	0.81 ± 0.99	0.80 ± 1.12

^(a)^*Lachnospiracea incertae sedis.* The values are the mean ± standard deviation. No significant difference was observed in either inter−group comparison (by repeated measures ANOVA followed by unpaired *t*-test) or intra-group comparison (by Dunnett’s multiple comparison test) of each genus.

## Data Availability

The data that support the findings of this study are available from the corresponding author.

## Supplementary Materials

The following are available online at https://www.mdpi.com/article/10.3390/jof7090782/s1, Figure S1: The structure of GlcCer contained in *koji amazake*, Table S1: Background characteristics of the participants, Table S2: Illumina MiSeq sequencing information summary.
