# Supplementary material for: Intake of Koji Amazake Improves Defecation Frequency in Healthy Adults
Source: J Fungi (Basel). 2021 Sep 21;7(9):782. doi: 10.3390/jof7090782 (PMC8470246; doi:10.3390/jof7090782)
Supplement: Supplementary file 1 [file jof-07-00782-s001.zip › jof-1384547-supplementary/Supplemental File210901/Supplemental Table_1.pdf]

Table S1. Background characteristics of the subjects

Item	Observed value, means ± SD	
	Koji amazake n=22	Placebo n=22
Gender (male/female)	9/13	9/13
Age (years)	38.8 ± 14.0	40.6 ± 14.0
Height (cm)	165.3 ± 8.7	165.0 ± 9.9
Body weight (kg)	57.3 ± 10.0	57.7 ± 6.8
Body mass index (kg/m^2)	20.9 ± 2.6	21.1 ± 1.9
Systoric blood pressure（mmHg）	110.9 ± 13.9	112.0 ± 12.9
Triglyceride (mg/dL)	78.4 ±39.6	82.9 ± 29.2
Fasting blood glucose level (mg/dL)	88.8 ± 8.7	91.0 ± 7.6
Hemoflobin A1c (%)	5.33 ± 0.28	5.38 ± 0.32
Weekly defecation days (days)	3.95 ± 1.02	4.00 ± 1.04
Weekly defecation frequency (times)	4.68 ± 1.55	4.73 ± 1.63
