# Supplementary material for: Intake of Koji Amazake Improves Defecation Frequency in Healthy Adults
Source: J Fungi (Basel). 2021 Sep 21;7(9):782. doi: 10.3390/jof7090782 (PMC8470246; doi:10.3390/jof7090782)
Supplement: Supplementary file 1 [file jof-07-00782-s001.zip › jof-1384547-supplementary/Supplemental File210901/Supplemental Table_2.pdf]

Table S2. Illumina MiSeq sequencing information summary

Time point	Num. of sample	Total count	Mean	SD	Max	Min
0w		672,457	15,283	3,890	24,479	10,338
3w	44	2,034,436	46,237	9,036	77,124	31,283
5w		2,172,504	49,375	6,759	74,864	34,688

SD, standard deviation; Max, maximum: Min minimum.
