# Supplementary figures and images for: Intake of Koji Amazake Improves Defecation Frequency in Healthy Adults
Source: J Fungi (Basel). 2021 Sep 21;7(9):782. doi: 10.3390/jof7090782 (PMC8470246; doi:10.3390/jof7090782)

## Slide 1
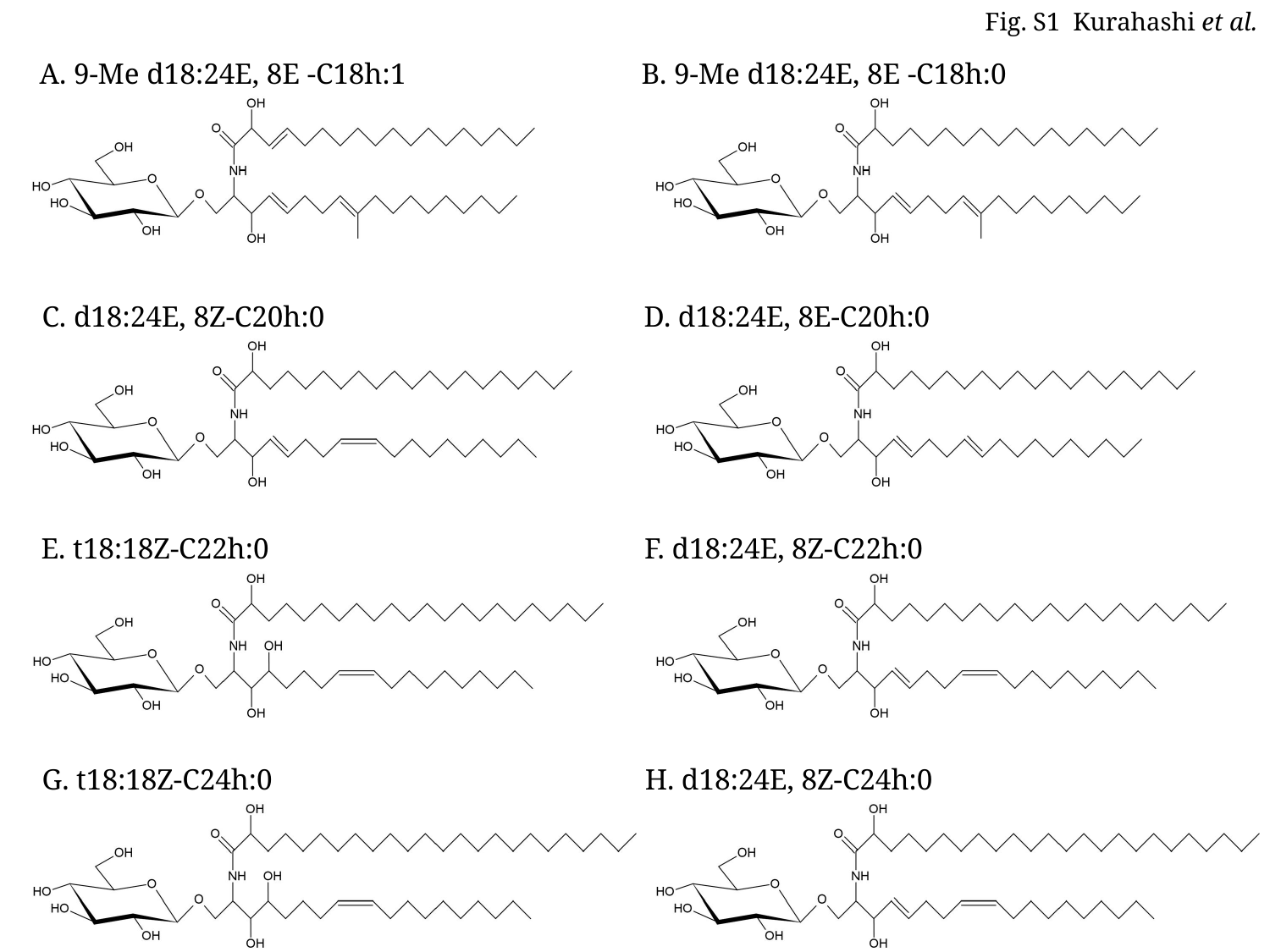

Fig. S1 Kurahashi et al.
A. 9-Me d18:24E, 8E -C18h:1
B. 9-Me d18:24E, 8E -C18h:0
C. d18:24E, 8Z-C20h:0
D. d18:24E, 8E-C20h:0
E. t18:18Z-C22h:0
F. d18:24E, 8Z-C22h:0
G. t18:18Z-C24h:0
H. d18:24E, 8Z-C24h:0

Supplement: Supplementary file 1 [file jof-07-00782-s001.zip › jof-1384547-supplementary/Supplemental File210901/Supplemental Figure_1.pptx]
